# Profiles of Sensitivity to Antibiotics and Heavy Metals in Strains of *Pseudomonas mendocina* Isolates from Leachate Pond

**DOI:** 10.3390/antibiotics14080781

**Published:** 2025-08-01

**Authors:** Aura Falco, Alejandra Mondragón-Quiguanas, Laura Burbano, Miguel Ángel Villaquirán-Muriel, Adriana Correa, Carlos Aranaga

**Affiliations:** 1 Departamento de Biología, Facultad de Ciencias, Universidad del Tolima, Ibagué 730006, Colombia; adfalcor@ut.edu.co; 2 Grupo de Investigación en Microbiología, Industria y Ambiente (GIMIA), Facultad de Ciencias Básicas, Universidad Santiago de Cali, Cali 760035, Colombia; alejandra.mondragon00@usc.edu.co (A.M.-Q.); laura.burbano00@usc.edu.co (L.B.); miguel.villaquiran00@usc.edu.co (M.Á.V.-M.); adriana.correa00@usc.edu.co (A.C.); 3 Laboratorio de Salud Pública Departamental, Secretaria Departamental de Salud del Valle del Cauca, Gobernación del Valle del Cauca, Cali 760045, Colombia; 4 Grupo de Investigación en Química y Biotecnología (QUIBIO), Facultad de Ciencias Básicas, Universidad Santiago de Cali, Cali 760035, Colombia

**Keywords:** *Pseudomonas mendocina*, leachates, antibiotics, tolerant, metals, aminoglycosides

## Abstract

**Background/Objetives**: Antimicrobial Resistance (AMR) is a multifaceted issue that the World Health Organization (WHO) identifies as one of the primary threats to global health for humans, animals, and the environment. In Colombia, AMR has been extensively studied at the hospital level; however, there are limited environmental studies, particularly concerning leachates from landfills. The objective of this study was to identify and determine the genetic relationships, as well as the sensitivity profiles to antibiotics and heavy metals, of ten *Pseudomonas mendocina* isolates from a leachate pond. **Methods**: Identification was conducted using MALDI-TOF (Matrix-Assisted Laser Desorption/Ionization Time-of-Flight), while genotyping was performed via rep-PCR. Antibiotic susceptibility to β-lactams, aminoglycosides, and quinolones was assessed using the Kirby-Bauer method. Additionally, sensitivity profile to heavy metals was evaluated using the broth microdilution technique. **Results**: Rep-PCR analysis indicated that 60% (*n* = 6/10) of the isolates exhibited a clonal relationship. Sensitivity testing revealed that 30% (*n* = 3/10) of the isolates displayed reduced sensitivity to aminoglycosides and β-lactams. Finally, the broth microdilution showed that 90% (*n* = 9/10) of the isolates were tolerant to copper sulfate. **Conclusions**: These results provide evidence that landfill leachates may serve as a potential reservoir for bacteria harboring antimicrobial resistance determinants.

## 1. Introduction

*Pseudomonas mendocina* is a Gram-negative bacterium belonging to the Pseudomonadaceae Family [1]. Its natural habitat is soil and water, and it was first isolated in Mendoza, Argentina [2]. Although *P. mendocina* has traditionally been considered an environmental bacterium, recent evidence suggests that it is an emerging opportunistic pathogen capable of causing serious infections. This is supported by an increasing number of clinical case reports describing its involvement in various human infections [3], including endocarditis [4,5,6,7,8], ocular keratitis [9], meningitis [10], urinary tract infections (UTIs) [11], bacteremia [12,13,14,15], spondylodiscitis [16], and peritonitis in patients undergoing peritoneal dialysis [17]. In addition, it has been reported to be the causative agent of mastitis in cattle [18].

Beyond its pathogenic potential, *P. mendocina* has also been isolated from various contaminated environments, including residual wastewater [19] and landfill leachates, which are characterized by elevated organic and inorganic pollutant loads [20]. Leachate ponds form due to the percolation of water through solid waste [21], a process that is exacerbated by global population growth and increasing waste generation [22]. These leachates often contain trace levels of antibiotics and heavy metals, posing risks to surface and groundwater quality and, consequently, to human, animal, and environmental health, consistent with the One Health approach [23].

Several studies have documented the presence of antibiotics in landfill leachates. Wu et al. [24] identified sulfonamides, tetracyclines, macrolides, and chloramphenicol in landfills in Shanghai, China. Similarly, You et al. [25] reported fluoroquinolones and β-lactams in landfills across seven megacities in China: Shanghai, Beijing, Guangzhou, Shenzhen, Tianjin, Chongqing, and Nanjing. Threedeach et al. [26] found *Escherichia coli* isolates from Thailand landfill leachates resistant to aminoglycosides, quinolones, chloramphenicol, sulfonamides, and β-lactams. *P. mendocina* has also been isolated from effluents of the wastewater treatment plant in Nanjing, China [27], as well as from hospital wastewater in Ghana and India, where multidrug-resistant strains have been identified [28].

In Colombia, two reports have documented antibiotic-resistant bacteria isolated from landfill leachates. One report identified β-lactam-resistant *Enterobacterales* [29], while the other found isolates of *Vibrio metschnikovii* and *Vibrio injensis* that were resistant to both β-lactams and aminoglycosides [30]. These findings confirm that landfill leachates can serve as reservoirs for pathogenic bacteria that are resistant to antibiotics.

In contrast to antibiotics, heavy metals are non-degradable and accumulate in the environment through various anthropogenic activities such as mining, metal processing, and the disposal of electronic waste. These elements also enter landfills through industrial and household waste, including medical and veterinary products that use metals as antimicrobial agents. Common heavy metals found in landfill environments include lead, mercury, cadmium, and chromium, known for their toxicity to humans and animals [31].

The environment contaminated with antibiotics and heavy metals waste exerts evolutionary pressure that favors the selection of antimicrobial-resistant bacteria [32]. From an epidemiological perspective, the situation becomes even more concerning when resistance determinants are located on Mobile Genetic Elements (MGE), such as plasmids or integrons, which facilitate Horizontal Gene Transfer (HGT) [33]. *P. mendocina* has demonstrated a capacity to tolerate heavy metals and participate in bioremediation processes. Chong et al. [34] conducted a genomic analysis of *P. mendocina* strain S5.2, which was isolated from soil in France. The authors suggest that this strain may have the potential to tolerate heavy metals, as they detected genes that are part of the *mer* operon, along with others that encode P-type ATPases, which are known for their roles in ionic homeostasis and biotolerance to heavy metal ions such as Cu^2+^, Cd^2+^, Zn^2+^, and Ag^+^ [34].

Resistance to heavy metals and antibiotics is often interconnected through shared mechanisms such as efflux pumps, reduced membrane permeability, and co-selection via MGE [35]. The One Health framework emphasizes the interconnectedness of human, animal, and environmental systems. In this context, the World Health Organization (WHO) recognizes antimicrobial resistance (AMR) as one of the most urgent global health threats, projecting potential healthcare costs of up to US$1 trillion annually by 2050 if current trends continue [36].

Although infections caused by *P. mendocina* remain rare, understanding the dynamics of resistance in emerging environmental species is essential to anticipate and mitigate their potential clinical and economic impact. Therefore, the objective of this study was to identify and determine the genetic relationships, as well as the susceptibility profiles to antibiotics and heavy metals, in *P. mendocina* isolates from landfill leachates.

## 2. Results

### 2.1. Isolation and Identification of Pseudomonas

A total of ten non-lactose fermenting isolates growing on Cetrimide Agar were obtained. All isolates were confirmed as Gram-negative bacilli and identified as *P. mendocina* using MALDI-TOF (VITEK^®^ MS bioMérieux, Durham, NC, USA), yielding high-confidence scores (≥99.9%).

### 2.2. Antibiotic Sensitivity Profile in Pseudomonas mendocina Isolates

Among the ten isolates, 20% (*n* = 2/10) showed reduced sensitivity to aminoglycosides, distributed as follows: L3-12 exhibited diminished sensitivity to amikacin, while L3-3 showed reduced sensitivity to gentamicin (Table 1). Additionally, one isolate, L3-1 (Table 1), demonstrated reduced susceptibility to imipenem. Seventy percent (*n* = 7/10) of the isolates were sensitive to all antibiotics tested.

### 2.3. Genetic Relationship Between Pseudomonas mendocina Isolates

**Figure 1:**
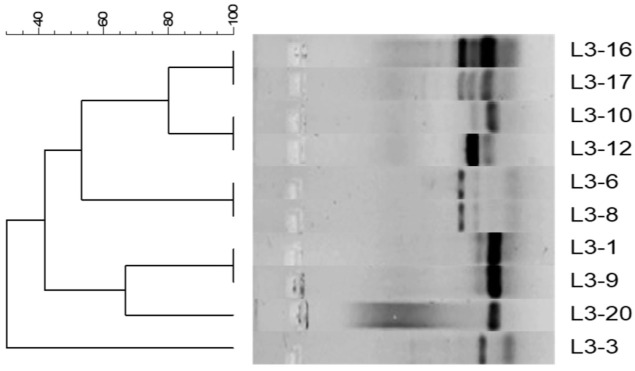
Rep-PCR profiles of ten *P. mendocina* isolates obtained from a leachate pond in Cali, Colombia. The band patterns were analyzed using Bionumerics (version 7.5, Applied Maths NV/Inc., Sint-Martens-Latem, Belgium), employing the binary coefficient Dice and the Unweighted Pair Group Method (UPGM) with adjustments for tolerance and optimization set at 1% to create the dendrogram. The positions of the bands were normalized using the 100 bp Ladder molecular weight marker (New England Biolabs^®^ Inc., Ipswich, MA, USA) as an external reference standard.

In the analysis of rep-PCR, indistinguishable band patterns can be observed between isolates L3-1 and L3-9, L3-6 and L3-8, and L3-16 and L3-17, indicating that these are clones. Furthermore, L3-20 is closely related to the clones L3-1 and L3-9. Similarly, L3-10 and L3-12 are closely related to L3-16 and L3-17 (Figure 1). Finally, L3-3 shows no relationship with the other isolates (Figure 1).

### 2.4. Conjugation Assays in Pseudomonas mendocina Isolates with Decreased Antibiotic Sensitivity

Conjugation assays were performed using the following donor strains: L3-12, which exhibited diminished sensitivity to amikacin (Figure 1), and L3-1, which showed reduced sensitivity to imipenem (Table 1). These strains could transfer DNA associated with resistance to the aforementioned antibiotics to the recipient strain *E. coli* J-53 through the process of conjugation (Table 2). This capability was determined by the growth of transconjugants on Trypticase Soy Agar (TSA) supplemented with rifampicin, amikacin, and imipenem, respectively. In contrast, isolate L3-3 was unable to conjugate with *E. coli* J-53 under the tested conditions.

### 2.5. Heavy Metal Susceptibility Profiles of Pseudomonas mendocina Isolates

Minimum Inhibitory Concentrations (MIC) testing of heavy metal salts indicates that 90% (*n* = 9/10) of the isolates were tolerant to CuSO_4_, 60% (*n* = 6/10) to NiSO_4_, and 40% (*n* = 4/10) to CdCl_2_ (Table 3). In addition, 90% (*n* = 9/10) of the isolates demonstrated tolerance to Pb(NO_3_)_2_ comparable to the control strain *P. aeruginosa* ATCC 27853_,_ while 50% (*n* = 5/10) were tolerant to ZnSO_4_ (Table 3). Furthermore, all isolates (*n* = 10/10) exhibited lower tolerance to CoCl_2_ compared to the control strain, and none showed tolerance to HgCl_2_ under the tested conditions (Table 3).

## 3. Discussion

In this study, ten isolates of non-fermenting Gram-negative bacteria were recovered from landfill leachate and identified as *P. mendocina* using MALDI-TOF mass spectrometry. To the best of our knowledge, this is the first report of *P. mendocina* in landfill leachates in Colombia.

The antibiotics tested in this study were selected based on their common use in Colombian healthcare settings for the treatment of Gram-negative infections, including those potentially caused by *Pseudomonas* spp. Although CLSI guidelines are designed primarily for *P. aeruginosa*, limited guidance is available for *P. mendocina*. Therefore, the antibiotics chosen provide a practical and clinically relevant approach for evaluating environmental resistance patterns. According to the results, 20% of the isolates (*n* = 2/10) exhibited reduced sensitivity to aminoglycosides (amikacin and gentamicin), while 10% (*n* = 1/10) demonstrated reduced sensitivity to imipenem, a carbapenem (Table 1). These findings align with previous clinical reports. In accordance with the 21 case studies published to date, *P. mendocina* is capable of causing infections in humans, the most common of which is endocarditis [4,5,6,7,8]. Ioannou et al. [3] reported that, among these 21 cases, resistance to ampicillin was 80%, to cotrimoxazole was 33%, to carbapenems was 10%, and to fourth-generation cephalosporins was 53.3%, while resistance to aminoglycosides was 20%, to quinolones was 33.3%, and to colistin was 6.7%. According to various reports, the most common antibiotics for treating *P. mendocina* infections are fluoroquinolones and third-generation cephalosporins [3,13,17]. In our study, the isolate with reduced sensitivity to imipenem is noteworthy, as Fang et al. [37] have reported *P. mendocina* isolates carrying the genes that encode for carbapenemases, NDM-1 and IMP-1, and demonstrated their transferability via plasmid conjugation.

Although the presence of transconjugants in our study suggests HGT of antibiotic resistance determinants, the absence of plasmid extraction or molecular confirmation (e.g., PCR or sequencing) is a limitation. However, the acquisition of reduced susceptibility to β-lactam antibiotics by *E. coli* J-53, a rifampicin-resistant strain naturally susceptible to β-lactams, supports the hypothesis that resistance genes were mobilized via conjugative plasmids. Moreover, the fact that the transconjugants mirrored the resistant profile of the donor strains further indicates successful horizontal transfer. Future research should include plasmid profiling and molecular characterization to confirm these findings and clarify the underlying mechanisms.

In terms of heavy metal tolerance, 90% (*n* = 9/10) of isolates were tolerant to CuSO_4_, 60% (*n* = 6/10) to NiSO_4_, and 40% (*n* = 4/10) to CdCl_2_ (Table 2). This tolerance is consistent with the metabolic versatility of bacteria belonging to the genus *Pseudomonas*, which confers an evolutionary advantage in contaminated environments. Consequently, these microorganisms have been proposed as potential agents for the bioremediation of metal-polluted soils. For instance, an isolate of *P. mendocina* collected from the copper mines of Khetri, in the Jhunjhunu district of Rajasthan, India, demonstrated tolerance to arsenic [38]. Similarly, Carrazca et al. [39] reported arsenic tolerance in *P. mendocina* originating from mines in Durango, Mexico.

The tolerance to heavy metals is associated with various mechanisms, including biosorption, precipitation of heavy metals, efflux pumps, and the production of chelating ligands and siderophores. In *P. mendocina*, as in other heavy metal-resistant bacteria [38,39,40], specific genes may be present in the genome that encode proteins responsible for resistance to these metals. These genes can be transferred between bacteria through the process of HGT [40]. However, there are no previous reports of *P. mendocina* strains exhibiting tolerance to copper sulfate and nickel sulfate; therefore, this study provides the first report of such traits.

When examining resistance to both antibiotics and heavy metals, our results partially align with previous findings. For instance, *P. mendocina* isolates from the Almendares River in Cuba were reported to be resistant to chromium [41], while others from contaminated soil exhibited tolerance to mercury, lead, and cadmium, and showed potential to resist multiple drugs through efflux pumps [34]. These findings reinforce the role of environmental bacteria as reservoirs of antimicrobial resistance genes, expanding the scope of concern beyond clinical environments.

One of the limitations of this study is the absence of a chemical characterization of the landfill leachate samples. Future studies should include chemical analyses of the leachates to better understand the selective pressures shaping the resistome of environmental bacteria and to strengthen the ecological interpretation of resistance mechanisms.

Although this study demonstrated HGT of antibiotic resistance from *P. mendocina* to *E. coli* J-53, no such transfer was observed for heavy metal tolerance. Despite testing donor strains that exhibited phenotypic tolerance to copper, nickel, and cadmium, the recipient strain showed no change in its metal resistance profile after conjugation. This may be due to several factors. First, metal resistance genes may be chromosomally encoded, making them non-transferable by conjugation. Second, even if plasmid-borne, these genes may not be expressed in *E. coli* due to interspecies regulatory incompatibilities, such as mismatched promoters, transcription factors, or post-transcriptional mechanisms. These barriers to heterologous gene expression across phylogenetically distant species have been documented [42,43]. Therefore, the lack of observable gene transfer does not rule out the potential mobility of heavy metal resistance determinants. Future studies should consider the whole genome or plasmid sequencing to determine the location of resistance genes.

Interestingly, 70% (*n* = 7/10) of the *P. mendocina* isolates were phenotypically sensitive to all tested antibiotics; however, several of these exhibited heavy metal tolerances. This is particularly concerning in the context of landfill leachates, which host diverse bacterial populations, including potential pathogens. The presence of mobile resistance genes, even in isolates with low phenotypic resistance, suggests an environmental risk of horizontal gene dissemination. From a One Health perspective, these findings emphasize the importance of monitoring environmental reservoirs of resistance beyond traditional clinical definitions of multidrug resistance.

To further investigate the potential for plasmid-mediated resistance, we performed conjugation experiments with isolates L3-1, L3-3, and L3-12, which showed reduced sensitivity to aminoglycosides and imipenem. This study represents the first report of conjugation between isolates of *P. mendocina* and *E. coli*. Such events have significant environmental implications [33], as bacteria can act as reservoirs of resistant genes with the potential to impact environmental, human, and animal health [44,45,46].

**Figure 2:**
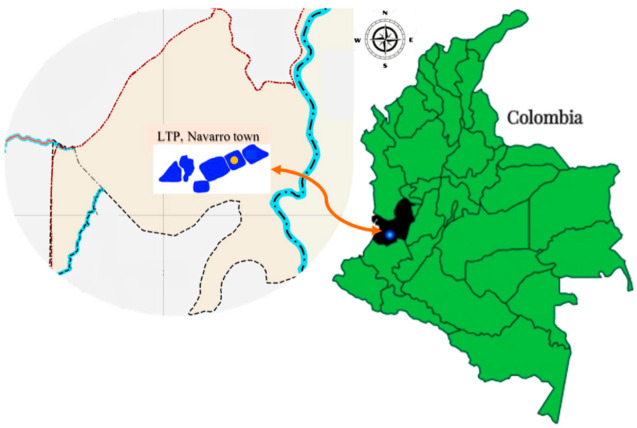
The location of the leachate treatment plant (LTP) in Navarro town, Santiago de Cali municipality (blue dot), Valle del Cauca department Table (black), and the sampling location: Lagoon 3 (yellow dot).

Rep-PCR analysis revealed indistinguishable banding profiles between isolates of *P. mendocina* L3-1 and L3-9, L3-6 and L3-8, and L3-16 and L3-17, indicating that these isolates are clones (Figure 2). Additionally, L3-20 appeared closely related to the clones L3-1 and L3-9, while L3-10 and L3-12 were genetically similar to L3-16 and L3-17 (Figure 2). Despite their high genetic similarity, some of these clones exhibited phenotypic differences in resistance to antibiotics and heavy metals. These differences may be due to the acquisition or loss of MGE, such as plasmids, integrons, and transposons, or point mutations in chromosomal resistance genes [47]. Conversely, other clonal pairs, such as L3-6/L3-8 and L3-16/L3-17, showed concordance at both genotypic and phenotypic levels.

These findings highlight the complexity of resistance dynamics in environmental bacteria and suggest that shared genetic features may result from common selective pressures in similar ecological niches [48]. To date, no previous studies have applied rep-PCR genotyping to *P. mendocina*. While Pulse Field Gel Electrophoresis (PFGE) remains the gold standard technique for bacterial genotyping, it is more expensive, labor-intensive, and lacks global standardization. Rep-PCR, although less costly, also lacks interlaboratory comparability [49], and no Multi-Locus Sequence Typing (MLST) schemes are currently available for this species. Whole genome sequencing would provide a more robust and comparative analysis, representing a limitation of this study.

In recent years, there has been an increasing number of reports regarding *P. mendocina*’s impact on human and animal health, indicating that it may be classified as an emergent opportunistic pathogen [50]. Therefore, identification, molecular characterization, and genetic analysis of environmental isolates are essential for understanding the genetic diversity of this species and its potential to disseminate resistance traits. Moreover, deciphering the mechanisms by which these bacteria survive in contaminated environments will be critical for addressing the global challenges of antimicrobial resistance and environmental pollution.

## 4. Materials and Methods

### 4.1. Sampling and Collection Site of Leachate Sample

This is a descriptive study employing purposive and intentional sampling, along with selective and differential cultivation methods, to promote the growth of presumptive *Pseudomonas* spp. Leachate samples were collected from Lagoon 3 of a Treatment Plant Landfill (TPL) in the Municipality of Navarro (3°23′13.8″ N, 76°29′7.5″ W), Valle del Cauca, Colombia. The TPL was established in 2008 following the closure of a municipal landfill used for solid waste disposal. It consists of eight lagoons; five are treated using physicochemical methods, while the remaining three, including Lagoon 3, remain untreated (Figure 2).

### 4.2. Isolation and Identification of Pseudomonas

A total of 300 mL of leachate was collected from Lagoon 3 and filtered in three 100 mL aliquots using cellulose ester membrane (0.45 µm pore membrane; Merck Millipore, Darmstadt, Germany) with vacuum filtration equipment. The membranes were then placed on MacConkey and Cetrimide agar plates (Merck Millipore, Darmstadt, Germany) and incubated at 37 °C for 18–20 h. Colonies with presumptive phenotype of *Pseudomonas* were subcultured on Trypticase Soy Agar (Merck Millipore, Darmstadt, Germany) for purification. The presumed colonies of *Pseudomonas* were identified using MALDI-TOF MS (VITEK^®^ MS bioMérieux, Durham, NC, USA), following the manufacturer’s protocol.

### 4.3. Determination of Antibiotic Susceptibility Profiles in P. mendocina Isolates

Antibiotic susceptibility was determined using the Kirby-Bauer disk diffusion method, in accordance with the Clinical and Laboratory Standards Institute guidelines [51]. Isolates were first subcultured on Mueller-Hinton (MH) agar (bioMérieux, Durham, NC, USA) and suspended in a saline solution (SS, 0.85%) to a turbidity equivalent to 0.5 McFarland. The bacterial suspension was then plated en masse onto MH agar plates using a sterile swab. Antibiotic discs were placed on the agar surface, and plates were incubated at 37 °C for 18 to 20 h.

The antibiotics tested included: Ceftazidime (30 µg), Cefotaxime (30 µg), Ciprofloxacin (5 µg), Ertapenem (10 µg), Imipenem (10 µg), Amikacin (30 µg), and Gentamicin (10 µg) (Oxoid, Basingstoke, UK). The antibiotics represent three major antimicrobial classes, such as β-lactams, fluoroquinolones, and aminoglycosides, and were selected based on their frequent use in Colombia for treating Gram-negative infections. Inhibition zones were measured with a vernier caliper and interpreted according to the following M-100 CLSI guidelines [51]. As no species-specific breakpoints exist for *P. mendocina*, results were interpreted based on *P. aeruginosa* standards. The strain *P. aeruginosa* ATCC 27853 was used as the quality control strain.

### 4.4. Sensitivity Profile of P. mendocina Isolates to Heavy Metals

The microdilution method was used to evaluate tolerance to heavy metals. Trypticase Soy Broth (TSB; Merck Millipore, Darmstadt, Germany) was supplemented with the following salts: lead nitrate (12.5 mM), cobalt chloride (0.625 mM), zinc sulfate (12.5 mM), nickel sulfate (6.25 mM), copper sulfate (6.25 mM), cadmium chloride (6.25 mM), and mercury (II) chloride (6.25 mM).

Each isolate was grown overnight in 2 mL of TSB at 37 °C. The cellular density was adjusted to 4–6 × 10^6^ cells/mL, and 0.1 mL of this suspension was inoculated into 96-well microplates. An equal volume (0.1 mL) of TSB supplemented with the respective metal salt was added to the wells. Controls include (i) a sterile control (medium + metal only), (ii) a medium control (medium without bacteria or metal), and (iii) a growth control (medium + bacteria, without metal). Microplates were incubated at 37 °C for 24 h. Afterward, 30 μL of resazurin (0.04%) was added to each well and incubated at 37 °C for an additional 4 h. The Minimum Inhibitory Concentration (MIC) was defined as the lowest visible concentration of metal that prevented a color change from blue to pink, indicating inhibited metabolic activity. Due to the absence of established concentrations of universally accepted metals for defining tolerance or resistance, the MIC was defined as the lowest concentration of metal that inhibited visible growth after an incubation period of 72 h at the optimal growth temperature. *Pseudomonas aeruginosa* ATCC 27853 was used as the control strain, as no control was available for the species *P. mendocina*.

### 4.5. DNA Extraction of P. mendocina Isolates

DNA was extracted from *P. mendocina* isolates using a boiling lysis method. A single colony from TSA was resuspended in 100 μL of sterile distilled water and heated at 100 °C for 10 min. Lysates were centrifuged at 13,000 rpm for 5 min, and the supernatant was used as a DNA template for PCR. The quality of extracted DNA was verified by PCR amplification of the gene encoding the 16S ribosomal RNA subunit using universal primers U1 (5′-CCAGCAGCCGCGGTAATACG-3′) and U2 (5′-ATCGG(C/T)TACCTTGTTACGACTTC-3′) [52].

### 4.6. Amplification by PCR of Repetitive Extragenic Palindromic Sequences (Rep-PCR)

The genetic relationships among *P. mendocina* isolates were determined using polymerase chain reaction (PCR) of repetitive extragenic palindromic sequences (rep-PCR). Primers REP1 (5′-IIIGCGCCGICATCAGGC-3′) and REP2 (5′-ACGTCTTATCAGGCCTAC-3′) [53] were used at 0.5 µM each. The PCR mix included 10% DNA template, 100 µL of 2X PCR master mix (CorpoGen, Bogotá, Colombia, OP-TBM-00006) for a final 1X concentration, and ultrapure water to adjust the volume.

PCR was performed in a thermocycler (Eppendorf Mastercycler personal, Z316391, Hamburg, Germany) using the following conditions: initial denaturation at 95 °C for 5 min, followed by 30 cycles of denaturation at 94 °C for 1 min, annealing at 45 °C for 1 min, and extension at 72 °C for 8 min, with a final extension at 72 °C for 10 min [53]. As a negative control for the reaction, the volume of DNA was replaced with sterile water.

The criteria for interpreting the pattern generated by rep-PCR were established by Tenover et al. [54], who defined four categories of genetic relationships and epidemiological significance: (a) Indistinguishable—isolates exhibiting identical band patterns can be considered clones; (b) Closely related—band patterns that differ by two or three bands; (c) Possibly related—patterns showing four to six different bands; and (d) Unrelated—patterns differing by more than six bands. Band profiles were analyzed using Bionumerics software, version 7.5 (Applied Maths NV/Inc., Sint-Martens-Latem, Belgium), employing Dice’s coefficient and the Unweighted Pair Group Method (UPGM) with optimization and tolerance set to 1% to create the dendrogram. The positions of the bands were normalized using the 100 bp Ladder molecular weight marker (New England Biolabs^®^ Inc., Ipswich, MA, USA) as an external reference standard.

### 4.7. Horizontal Gene Transfer via Conjugation

Conjugation assays were performed to evaluate the horizontal transfer of antibiotic resistance genes. Donor strains *P. mendocina* L3-1, L3-3, and L3-12 (OD_600nm_ 0.2), which showed reduced sensitivity to aminoglycosides and β-lactams (Table 1), were mixed with recipient strain *E. coli* J-53 (F^−^ *met pro* Rif^R^) [55] (OD_600nm_ 0.1) at a 1:10 donor-to-recipient ratio in TSB. The mixture was incubated at 37 °C for 24 h. Post-incubation, conjugation mixtures and serial dilutions (in 0.85% SS) were plated on TSA supplemented with rifampicin (25 μg/mL) and one of the following: amikacin (100 μg/mL), gentamicin (7 μg/mL), or imipenem (1 μg/mL), to select and quantify transconjugants. Plates were incubated at 37 °C for 18-20 h, after which we assessed the growth of transconjugants. As positive control, *E. coli* 1646 carrying a conjugative plasmid with *bla*_KPC-2_ gene (conferring resistance to β-lactams) was included.

The frequency of transfer (FT) was calculated as follows:

$$\mathrm{FT}=\frac{\mathrm{Titre}\mathrm{of}\mathrm{transconjugants}}{\mathrm{Titre}\mathrm{of}\mathrm{donnors}}$$

## 5. Conclusions

This study demonstrates that leachates serve as a potential reservoir for *P. mendocina,* some of which exhibit reduced sensitivity to antibiotics and tolerance to heavy metals. This is the first report in Colombia to characterize *P. mendocina* isolates from leachates in terms of their antibiotic susceptibility, metal tolerance, and genetic features. Although the overall frequency of resistance, the presence of genetically related strains harboring conjugative plasmids highlights the potential for HGT. These findings underscore the need to monitor microbial communities in waste environments, as they may contribute to the environmental dissemination of resistance genes. The results may inform future surveillance strategies and environmental policies aimed at mitigating the spread of antimicrobial resistance.

## Figures and Tables

**Table 1:** Antimicrobial susceptibility profiles of *Pseudomonas mendocina*.

Kirby-Bauer Test (μg/mL) by Antibiotic/Interpretive Categories							
Strain ID	AK ^1^	CN ^1^	CAZ ^2^	IMP ^2^	CTX ^2^	ETP ^2^	CIP ^3^
L3-1	≤2 (S)	≤1 (S)	≤1 (S)	16 (I)	≤1 (S)	≤1 (S)	≤0.25 (S)
L3-3	≤2 (S)	14 (I)	≤1 (S)	≤0.25 (S)	≤1 (S)	≤1 (S)	≤0.25 (S)
L3-6	≤2 (S)	2 (S)	2 (S)	0.5 (S)	2 (S)	2 (S)	≤0.25 (S)
L3-8	≤2 (S)	≤1 (S)	2 (S)	≤0.25 (S)	2 (S)	2 (S)	≤0.25 (S)
L3-9	≤2 (S)	≤1 (S)	2 (S)	≤0.25 (S)	2 (S)	2 (S)	≤0.25 (S)
L3-10	≤2 (S)	≤1 (S)	2 (S)	≤0.5 (S)	2 (S)	2 (S)	≤0.25 (S)
L3-12	16 (I)	≤1 (S)	≤1 (S)	≤0.5 (S)	≤1 (S)	≤1 (S)	≤0.25 (S)
L3-16	≤2 (S)	≤1 (S)	≤1 (S)	0.5 (S)	≤1 (S)	≤1 (S)	≤0.25 (S)
L3-17	≤2 (S)	≤1 (S)	≤1 (S)	0.5 (S)	≤1 (S)	≤1 (S)	≤0.25 (S)
L3-20	≤2 (S)	≤1 (S)	≤1 (S)	≤0.25 (S)	≤1 (S)	≤1 (S)	≤0.25 (S)

Abbreviations: R: Susceptible; I: Intermediate; R: Resistant; Ak: Amikacin; CN: Gentamicin; ETP: Ertapenem; CTX: Cefotaxime; CAZ: Ceftazidime; IMP: Imipenem; CIP: Ciprofloxacin; ^1^ Aminoglycosides; ^2^ β-lactams; ^3^ Fluoroquinolones.

**Table 2:** Donor and Recipient Strain Titers and Transfer Frequency.

Titers of Donor and Recipient Strains and Frequency of Transfer	
Strain	Transfer Frequency
*E. coli* J-53 (recipient)	Does not apply
*E. coli* 1646 (donor, control)	0.53
*P. mendocina* L3-1 (donor)	6.4 × 10^4^
*P. mendocina* L3-3 (donor)	There were no transconjugants
*P. mendocina* L3-12 (donor)	0.03

**Table 3:** Minimum Inhibitory Concentration (MIC) of Heavy Metals in *P. mendocina* Isolates.

Minimum Inhibitory Concentrations for Metals (mM)							
Strain	Pb(NO_2_)_3_	CoCl_2_	ZnSO_4_	NiSO_4_	CuSO_4_	CdCl_2_	HgCl_2_
*P. aeruginosa* ATCC 27853	>50	6.25	>12.5	>6.25	0.19	>6.25	6.25
L3-1	>50	2.5	12.5	12.5	0.19	6.25	N/G
L3-3	25	2.5	12.5	12.5	>3.125	1.56	N/G
L3-6	>50	2.5	>12.5	12.5	1.56	>6.25	N/G
L3-8	>50	2.5	>12.5	6.25	1.56	>6.25	N/G
L3-9	>50	2.5	>12.5	6.25	0.39	6.25	N/G
L3-10	>50	2.5	6.25	6.25	>3.125	6.25	N/G
L3-12	>50	2.5	3.125	3.125	>3.125	6.25	N/G
L3-16	>50	5	6.125	12.5	>3.125	>6.25	N/G
L3-17	>50	5	>6.25	12.5	>3.125	>6.25	N/G
L3-20	>50	5	>6.25	12.5	>3.125	6.25	N/G

Abbreviations: MIC, minimum inhibitory concentration; N/G, No growth; Pa, *Pseudomonas aeruginosa*; Pb(NO_2_)_3_, Lead nitrate; CoCl_2_, Cobalt chloride; ZnSO_4_, Zinc sulphate; NiSO_4_, Nickel sulphate; CuSO_4_, Copper sulphate; CdCl_2_, Cadmium chloride; HgCl_2_, Mercuric chloride.

## Data Availability

The data used to support the findings of this study are included within the article and are available from the corresponding author upon request.
